# Editorial: The roles of micro-RNAs in neuroendocrine systems

**DOI:** 10.3389/fendo.2024.1349804

**Published:** 2024-01-24

**Authors:** Lourdes Mounien, Kazuaki Nakamura, Fatiha Chigr, Fumihiko Maekawa

**Affiliations:** ^1^ Aix Marseille Université, C2VN, INRAE, INSERM, Marseille, France; ^2^ National Research Institute for Child Health and Development, Tokyo, Japan; ^3^ Université Sultan Moulay Slimane, Béni Mellal, Morocco; ^4^ National Institute for Environmental Studies (NIES), Tsukuba, Japan

**Keywords:** microRNA, neuroendocrine axis, neuroendocrine tumor, cancer, neuroendocrinologicalmarkers

Understanding the molecular mechanisms that regulate neuroendocrine systems is an exciting area of research, leading to a better comprehension of physiology and pathophysiology. In this context, evidence suggests that microRNAs, short non-coding RNAs, are involved in the regulation of hormone synthesis and release. This Research Topic, “*The Roles of MicroRNAs in Neuroendocrine Systems*”, focuses on the latest discoveries and advances in the field of relationships between microRNAs and the neuroendocrine processes. The selected articles cover various aspects of microRNA-based regulation and explore its effect on neuroendocrine responses in both healthy and pathophysiological states.

## 1 MicroRNAs as neuroendocrine modulators

The chosen articles highlight the role of microRNAs as key modulators in neuroendocrine signaling cascades. These studies shed light on the fine-tuned regulation of hormone release and receptor expression which are crucial aspects of neuroendocrine systems.

## 2 Neuroendocrine dysfunction in pathological states

By means of different approaches, the studies presented in this Research Topic explore how dysregulation of microRNAs expression contributes to neuroendocrine dysfunction in various pathologies, such as adrenal or thyroid diseases (Hara et al., Mondin et al., Ma et al.).

These findings offer critical insights into potential therapeutic aspects.

Interestingly, one of the articles in this Research Topic suggests that environment factors, such as endocrine disruptors like phthalates, could lead to a dysregulation of microRNA expression (Nazzari et al.).

## 3 Crosstalk between microRNAs and hormones

These articles provide an understanding of how microRNAs contribute to the dynamic regulation of hormone release. As illustrated in the literature and in the articles of this Research Topic, circulating microRNAs are identified as biomarkers of hormonal dysfunction and may be associated with the pathophysiological heterogeneity of several diseases (Hara et al.).

## 4 Therapeutic implications and future directions

The data presented in this Research Topic make it evident that microRNAs play a role beyond basic research. Particularly, the articles indicate potential therapeutic applications where microRNA-based interventions could impact neuroendocrine treatment.

In conclusion, the articles published in this Research Topic provide valuable insights into understanding the impacts of microRNAs on neuroendocrine regulation. This Research Topic not only enhances our scientific knowledge but also indicates a medical perspective where modulating microRNAs expression could revolutionize our approach of neuroendocrine disorders. Particularly, microRNA therapy holds promise for patients dealing with therapeutic or drug resistance which are critical issues in many diseases.

## Author contributions

LM: Writing – original draft, Writing – review & editing. KN: Writing – review & editing. FC: Writing – review & editing. FM: Writing – review & editing.

